# Editorial: Craniofacial Growth and Development: Novel Insights

**DOI:** 10.3389/fcell.2021.744711

**Published:** 2021-08-20

**Authors:** Erika Calvano Küchler, Rafaela Scariot, Christian Kirschneck

**Affiliations:** ^1^Department of Orthodontics, University Medical Centre of Regensburg, Regensburg, Germany; ^2^Department of Stomatology, Federal University of Paraná, Curitiba, Brazil

**Keywords:** craniofacial, genes, protein, tooth, dental development, craniofacial syndromes

The topic “Craniofacial Growth and Development: Novel Insights” is a Frontiers Research Topic aimed to provide an opportunity for researchers and clinicians from different perspectives and areas to publish recent advances in the understanding of craniofacial growth and development and craniofacial disorders. In addition, we also aimed to provide updated information regarding the etiological and risk factors involved in the development and establishment of the alterations and pathologies that affect the craniofacial complex. Here we present a variety of articles that contributed to the up-to-date literature in the craniofacial growth and development research field.

In this topic, Svandova et al. presented a review that aimed to discuss the formation and developmental specification of the odontogenic and osteogenic mesenchymes and to explore the mechanisms underlying condensation in both the odontogenic and osteogenic compartments. This same group also published a review focusing on the mechanisms behind the different fates of Meckel's cartilage (Svandova et al.)—Meckel's cartilage was first described by the German anatomist Johann Friedrich Meckel from his analysis of human embryos. In their work, the authors covered different aspects of the 200 years of the discovery of Meckel's Cartilage, including the early development of mammalian Meckel's cartilage and the diverse fates of this cartilage; how chondrocytes from Meckel's cartilage compare with chondrocytes of other types of cartilages and the proliferation, differentiation and maturation of chondroblasts; as well as the various molecular-biological aspects of Meckel's cartilage. The authors also explored some topics of clinical interest such as the patterning of the mandible and the consequences of defects in the development of Meckel's cartilage, once several human disorders are directly or indirectly connected with an abnormal Meckel's cartilage. Vasela et al. also explored mandibular development. They used an animal model (mice) and an in *vitro model* to investigate the caspase-12 in the context of mandibular development. The authors investigated, if caspase-12 participates in the modulation of osteogenic pathways and their study demonstrates caspase-12 expression in bone cells during craniofacial/mandibular development. A study from Sun et al. also used *in vivo* and *in vitro* models to investigate the role of Shh signaling on microvasculature in upper lip development. An animal model of Shh pathway antagonist-induced cleft lip was used to examine microvascular morphogenesis during normal and abnormal upper lip development and also an *in vitro* co-culture model to investigate Shh pathway perturbation on microvascular stability. The authors found a previously unrecognized role for Shh signaling in facial development and suggested microvascular morphogenesis as new focus for understanding normal and abnormal craniofacial development. Studies with animal models were also presented here in order to evaluate the role of bisphosphonates in bone healing. A study from Bonetto et al. used Wistar rats to investigate the effect of sodium alendronate on bone repair. The authors reported that alendronate had no effect on the parameters tested.

Another model explored in this issue was the zebrafish, which is an animal model that has been becoming more frequently used in studies to explore genes and environmental factors that are involved or affect craniofacial development. Yuan et al. tested, if ethanol-induced downregulation of microRNA-135a contributes to ethanol-induced apoptosis in neural crest cells by upregulating specific pathways. In their study, they observed that treatment with ethanol resulted in a significant decrease in microRNA-135a expression in both neural crest cells and zebrafish embryos. A review of the published zebrafish studies on environmental factors involved in the etiology of craniofacial malformations in humans including maternal smoking, alcohol consumption, nutrition and drug use, was also presented in this topic (Raterman et al.). The authors also highlighted the fact that the zebrafish is an excellent complementary model with high translational value to study complex interactions in the context of craniofacial development, such as gene-environmental interactions.

In the current topic, studies with human samples were also presented. Three studies investigated oral cleft patients (Alam and Alfawzan; Lou et al.; Latief et al.). Alam and Alfawzan investigated morphological characteristics associated with non-syndromic oral clefts and they found that Sella turcica bridging pattern is different in oral cleft and non-cleft patients and differences in the Sella turcica are also observed among cleft subtypes. This could indicate that morphological alterations related to oral clefts also involve other craniofacial structures. Lou et al. on the other hand, presented a study that used a case-control design to explore genetic variants associated with non-syndromic oral cleft. Genomic DNA from a large sample (2,027 cleft lip with or without cleft palate cases and 1,843 controls) was used to investigate genetic variants in 23 autophagy pathway genes. The authors identified that the genetic variant rs2301104 in Hypoxia-inducible factor 1a (HIF1A) gene contributed to the risk of non-syndromic oral cleft. The authors hypothesized that decreased expression of HIF1A might break the oxygen homeostatic response during embryonic development, thus inhibit autophagy and further exacerbate susceptibility to some developmental anomalies, including non-syndromic oral cleft. Martinelli et al. presented an overview regarding non-syndromic cleft palate exploring the known genetic and environmental risk factors.

Human samples were also used to explore developmental dental alterations. A pathological mineralization of the tooth structures, called pulp stones, was investigated in a case-control study design. These are characterized calcifications observed in the pulp chamber and highly frequent in the general population. The authors explored for the first time some salivary parameters and observed that patients with pulp stones have sialometric and sialochemical alterations.

A mini-review from Funato explored molecular aspects giving new insights into cranial synchondrosis development. The synchondroses form via endochondral ossification in the cranial base and play a critical role as an important growth center for the neurocranium. Abnormalities in the synchondroses affect cranial base elongation and the development of adjacent areas, including craniofacial bones. Early ossification and/or malformation of cranial synchondroses can induce the fusion of adjacent bones and subsequent cranial anomalies, like microcephaly and midface hypoplasia (Funato). In this review the development of cranial synchondroses and its regulation by the signaling pathways and transcription factors was presented, showing the differences between the intersphenoid and spheno-occipital synchondrosis.

Briefly, this Research Topic provided an opportunity for researchers and clinicians from different perspectives and areas to discuss recent advances in the understanding of craniofacial growth and development. It also will provide readers with new insights and different viewpoints to stimulate further investigations in this broad research field. This Research Topic also achieved its initial aim to have different areas contemplated, such as developmental biology, human genetics, craniofacial syndromes, cleft lip and palate and dental research, coming together in this article collection.

## Author Contributions

EK, RS, and CK contributed equally in the conception and written. All authors contributed to the article and approved the submitted version.

## Conflict of Interest

The authors declare that the research was conducted in the absence of any commercial or financial relationships that could be construed as a potential conflict of interest.

## Publisher's Note

All claims expressed in this article are solely those of the authors and do not necessarily represent those of their affiliated organizations, or those of the publisher, the editors and the reviewers. Any product that may be evaluated in this article, or claim that may be made by its manufacturer, is not guaranteed or endorsed by the publisher.

